# The dihydropyridine LA1011 modulates multiple Hsp90—co-chaperone interactions relevant to Alzheimer’s disease

**DOI:** 10.1016/j.cstres.2025.100131

**Published:** 2025-12-03

**Authors:** Xavier Jeanne, Jasmeen Oberoi, Mark S. Roe, Matthias Baud, John Spencer, Zsolt Torok, Laszlo Vigh, Chrisostomos Prodromou

**Affiliations:** 1 Department of Biochemistry and Biomedicine, University of Sussex, Brighton BN1 9QG, UK; 2 Genome Damage and Stability Centre, School of Life Sciences, University of Sussex, Brighton BN1 9RQ, UK; 3 School of Chemistry and Chemical Engineering, University of Southampton, Southampton SO17 1BJ, UK; 4 Sussex Drug Discovery Centre, School of Life Sciences, University of Sussex, Brighton BN1 9QJ, UK; 5 LipidArt Research and Development Ltd, Temesvári Street 62, H-6726, Szeged, Hungary

**Keywords:** Hsp90, Co-chaperones, Proteostasis, Hsp90 allosteric compounds, Alzheimer’s disease

## Abstract

LA1011 (dimethyl 4-(4-Trifluoro-methyl-phenyl)-2,6-bis(2-dimethylamino-ethyl)-1-methyl-1-4 dihydropyridine-3-5-dicarboxylate dihydrochloride) has been shown to improve the prognosis of Alzheimer’s disease (AD) in an APPxPS1 mouse model. The target for LA1011 is the C-terminal domain of Hsp90, where it was shown previously to reduce the interaction between FKBP51 and Hsp90. FKBP51 is a Hsp90 co-chaperone that promotes the *trans* to *cis* isomerization of proline at multiple tau pSer/pThr-pro sites, thus preventing their dephosphorylation. Potentially this leads to the hyperphosphorylation of tau and the formation of neurofibrillary tangles that eventually lead to the development of AD. In this study, we demonstrate that LA1011 affects the FKBP51-mediated regulation of Hsp90 but also potentially modulates the regulation Hsp90 by the co-chaperones FKBP52, CHIP, Aha1, Hch1 and PP5. We also show that the co-chaperones HOP, CDC37 and Sgt1 appear to enhance mildly the binding of LA1011. In contrast, nucleotide alone or nucleotide with Aha1 or p23, which promote the closed conformation of Hsp90, reduce the affinity for LA1011. We conclude that LA1011 can modulate the regulatory landscape of the Hsp90 co-chaperone network, which in turn appears to improve the prognosis of Alzheimer’s disease.

## Introduction

**Fig. 1:**
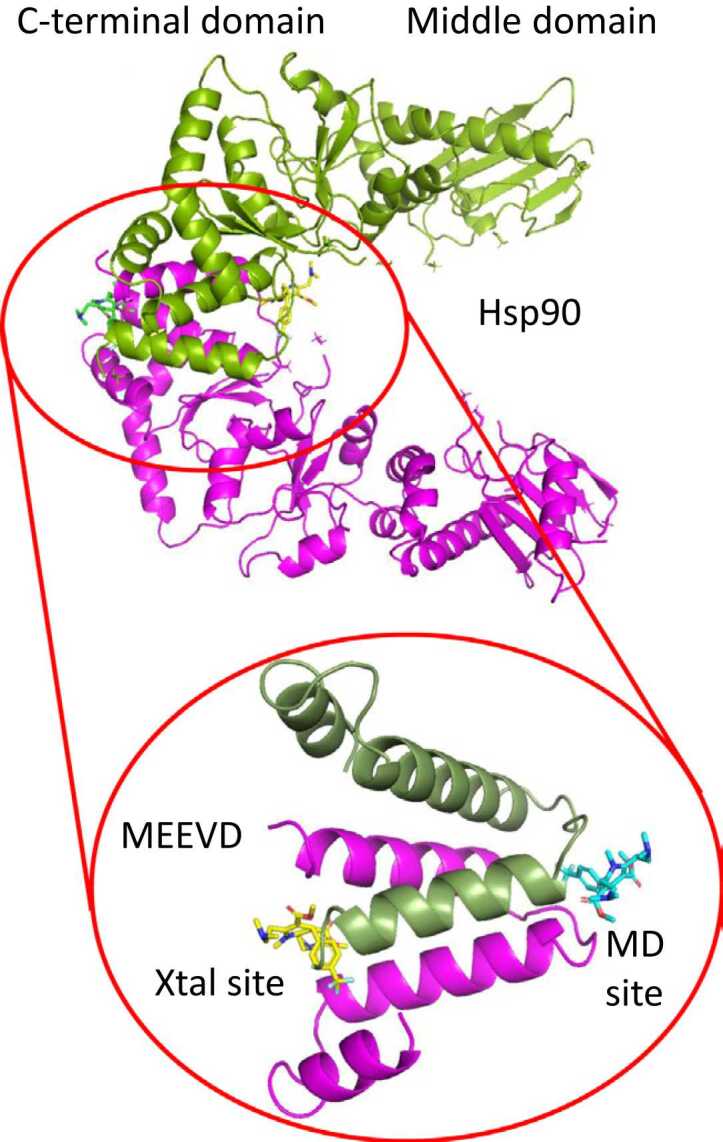
Molecular targets for LA1011 on the C-terminal domain of Hsp90. LA1011 is predicted to bind to the MD site (allosteric site) and has been seen to bind the Xtal site in crystallization studies. The lower panel shows the core of the C-terminal domain for clarity. Both binding sites occur within the C-terminal domain of Hsp90, at the ends of a four α-helical bundle, two helices from each Hsp90 protomer forming the core of the Hsp90 C-terminal domain.

Heat shock protein 90 (Hsp90) is a molecular chaperone that is involved in the regulation, maturation, and activation of a variety of client proteins.^1^, ^2^, ^3^ Imbalances in the Hsp90 chaperone system can provide mechanisms that lead to neurological diseases, such as Alzheimer’s disease (AD). The traditional approach for inhibiting Hsp90 has been to target its ATPase activity by competitive displacement of ATP from the N-terminal domain of the protein.^4^ This approach halts the Hsp90 chaperone cycle, and as a consequence, client proteins are then directed to the proteosome for degradation.^5^ While this approach targets clients that drive disease, to some degree all client proteins that have a significant requirement for Hsp90 chaperoning will be affected, thus eliciting toxicity and an antiapoptotic heat-shock-response. Consequently, this appears to have limited the success of such inhibitors for clinical use. In fact, to date only TAS-116 (pimitespib) has been approved for treatment of GIST cancers (gastrointestinal stromal tumor).^6^ Another limitation of this approach is that it does little to rebalance the chaperone system, so it is unable to address disease processes that result from dysfunctional proteostasis. However, there are a group of compounds, including LA1011, that interact with an allosteric site in the C-terminal domain of Hsp90 and act as activators of Hsp90 ATPase activity.^7^, ^8^, ^9^, ^10^, ^11^, ^12^, ^13^, ^14^, ^15^, ^16^ It was shown that the binding and unbinding of such compounds caused dynamic structural changes in the Hsp90 dimer at distances at the level of the N-terminal domain.^10^ They are proposed to elicit an asymmetric conformation in the Hsp90 dimer that is primed for sequential ATP hydrolysis.^15^, ^17^ Furthermore, such compounds have been shown to compete for binding to Hsp90 with the model client protein Δ131Δ, which is also a known Hsp90 ATPase activator. Competition for binding between C-terminal Hsp90-binding small molecules and client proteins suggests that disease-causing client proteins might be limited in their access to Hsp90 for activation, which consequently could hinder disease progression. This, therefore, led us to determine the structure of LA1011 in complex with Hsp90 to aid structure-based drug design. To this effect we identified two potential sites for the binding of LA1011, one using molecular dynamics simulations (which here we call the MD site) and another by co-crystallization with the C-terminal domain of Hsp90 (which here we call the Xtal site).^11^, ^12^ Our MD site appears to be the so-called allosteric site previously described.^7^, ^8^, ^9^, ^10^, ^13^, ^14^, ^15^, ^16^ The Xtal and MD site are nestled at the interface between the C-terminal dimerization domains, at directly opposite ends, and are essentially bound to the ends of a central core of a four α-helical bundle, formed by two α-helices from each protomer of the Hsp90 dimer (Figure 1). At each site a single molecule of LA1011 binds a dimer of Hsp90. To date, although LA1011 shows weak overall binding affinity to Hsp90 (previously the *K*d was measured at 13.5 μM^12^), LA1011 has a unique binding profile for Hsp90 that may allow it to modulate the chaperone network to combat disease driven by dysfunctional proteostasis.

The expression of co-chaperones in our brain changes as we age, but further changes in the AD brain can also be seen for some co-chaperones.^18^, ^19^ In fact, chaperone clusters representing groups of chaperones and co-chaperones have been shown to be repressed or induced in the aged and AD brain.^18^ Naturally, changes in specific co-chaperone expression may help promote the development of disease, while other changes could limit it. With such changes in the expression of co-chaperones in the AD brain, the question arises as to whether we can modulate the co-chaperone network to halt the development of AD? Compounds like LA1011 that effect an allosteric response on Hsp90 potentially offer an opportunity to modulate the chaperone network to disrupt the development of AD. To this effect, in an APPxPS1 AD mouse model LA1011 was able to orchestrate a number of changes that limited the progression of the AD.^20^ The authors observed a co-induction of the heat-shock-response, an increase in neuron number, decreases in neurofibrillary tangles and amyloid-beta (Aβ) plaques, and an increase in the dendritic spine density over those of untreated mice.^20^ With such profound effects, LA1011 is a prime lead candidate for the development of a clinical trial derivative.

Hsp90 not only stabilizes tau but is involved in regulating the status of its phosphorylation, which it does in concert with co-chaperones such as the immunophilin FKBP51, a peptidyl-propyl isomerase, and the phosphatase PP5.^19^, ^21^, ^22^ PP5 is responsible for removing phosphate from serine- and threonine-proline sites (pSer/pThr-Pro) of tau in a Hsp90 dependant fashion.^23^, ^24^ In contrast, FKBP51 catalyzes the isomerization of proline at pSer/pThr-Pro sites in tau from a *trans* to a *cis* conformation.^25^ The *cis* conformation of a pSer/pThr-Pro site is resistant to dephosphorylation by both PP2A and PP5,^26^ and potentially the overexpression of FKBP51 can lead to hyperphosphorylation of tau, which in turn aggregates into neurofibrillary tangles that ultimately drive the development of AD. PIN1 which can reverse the isomerization of *cis*-pSer/pThr-Pro sites towards the *trans* conformation, can help to limit this toxic cascade. However, in the AD brain, the levels of PIN1 are reduced, and there is an imbalance between the activities of PIN1 and FKBP51, that favor hyperphosphorylation of tau. Furthermore, lower levels of PIN1 may also increase the activity of GSK3β, which is associated with the phosphorylation of both amyloid precursor protein (APP) and multiple tau disease-associated phosphorylation sites.^27^, ^28^ Additionally, in the AD brain levels of p25 increase, which may in turn elevate CDK5 activity, which can promote the phosphorylation of the ^231^Thr-Pro site in tau, a known disease-associated event.^29^ Hence, in the AD brain there is a landscape of changes, including changes in the activity of the co-chaperone network, that favor the hyperphosphorylation of tau.^30^, ^31^ Recently, we proposed that elevated levels FKBP51 in the AD brain, above those seen in an aged brain, may drive the hyperphosphorylation of tau and that LA1011 could help reduce FKBP51 activity and thus restore proteostasis of tau.^11^, ^21^ We hypothesized that Aβ deposits in the brain, or other factors such as prolonged stress, induce the heat-shock-response protein FKBP51,^21^, ^32^ which in turn preserves neurotoxic sites of phosphorylation in tau that eventually drives the development of AD.^21^ In support of this idea, the preservation of neurotoxic tau by overexpression of FKBP51 as a Hsp90-dependent process has previously been shown.^30^ Furthermore, levels of FKBP51 are elevated in the AD brain,^30^, ^31^, ^33^ FKBP51 co-localizes with tau,^25^ and endogenous tau levels are reduced in double knockout mice for FKBP51.^30^, ^31^ Furthermore, we recently showed that LA101 reduces the affinity of FKBP51 for Hsp90, which could limit access of tau to FKBP51 activity. FKBP51 occupies the Xtal site, to which LA1011 binds, using a conserved hydrophobic motif found in helix-7 of its TPR domain. Based on a limited alignment, a ϕ/Yxxϕϕ motif is evident, where ϕ represents a hydrophobic residue and position 1 may also be tyrosine (Y).^11^ Following on from this observation, we hypothesized that preventing the Hsp90-dependent action of FKBP51 with LA1011 could perhaps reduce the phosphatase-resistant *cis* conformation of pSer/pThr-Pro bonds at multiple tau sites, and in turn limit or prevent the hyperphosphorylation of tau and the development of AD. Furthermore, the binding of LA1011 to the MD or allosteric site may reduce tau binding to the Hsp90-FKBP51 complex and therefore further reduce the effects of FKBP51 in stabilizing the phosphorylation status of toxic tau molecules. However, whatever the mechanism (and both may play a role), here we investigate the effect of LA1011 on the Hsp90 co-chaperone network to better understand how modulation of their mechanistic action might be beneficial to halting AD. We show that LA1011 modulates the Hsp90 chaperone cycle by altering the binding of multiple co-chaperones with Hsp90. Using isothermal titration calorimetry (ITC) and ATPase assays, we show that some co-chaperones (Aha1, Hch1, and PP5) can completely prevent the interaction of LA1011 with Hsp90, while others weaken it and yet others strengthen mildly the interaction of LA1011 with Hsp90. These findings highlight implications in terms of the selectivity of a future drug candidate and the potential of LA1011 as a therapeutic agent for AD, acting through its modulation of the Hsp90—co-chaperone network.

## Methods and materials

### Protein expression and purification

FKBP51 and FKBP51-Δ7He (lacking residues 401-457 of the extended 7th helix of the TPR domain) were a kind gift from D. Southworth (UCSF Weill Institute for Neurosciences) and were expressed with an N-terminal His6-tag.^34^ Yeast Hsp90, FKBP51, FKBP52, yeast Aha1, Hch1, p23, mouse CHIP, HOP, yeast Sti1, CDC37, full-length PP5, PP5-TPR domain (amino acid residues 1-177) and yeast Sgt1 (amino acid residues 1-280) were expressed and purified as previously described.^35^, ^36^, ^37^, ^38^, ^39^, ^40^, ^41^, ^42^ Essentially, proteins were purified using Talon affinity chromatography, followed by Superdex 75, Superdex 200 or Sephacryl 400 HR gel-filtration chromatography, as appropriate, and then finally with Q-Sepharose ion exchange (except for the TPR-PP5 domain). Proteins were dialyzed against 20 mM Tris pH 7.4 containing 5 mM NaCl and 1 mM EDTA, except for full-length PP5, which contained 1 μM MnCl_2_ and was devoid of EDTA.

### Hsp90 ATPase assays

ATPase assays using 2 μM Hsp90 were conducted as previously described using the lactate-dehydrogenase and pyruvate-kinase-linked assay.^12^ Briefly, 240 μM of LA1011 was used in assays as required. Co-chaperones were added to Hsp90 and Hsp90-LA1011 complex at a variety of concentrations ranging from 0.25 to 30 μM. Assays utilizing LA1011 were normalized by recalculating the activity for all data points so that the data points were converted to a percentage of the activity relative to the first data point, which lacked any addition of co-chaperone. The first data point was therefore 100% max Hsp90 activity. All assays were conducted at least in triplicate.

### Isothermal titration calorimetry

The heat of interaction was measured on an ITC_200_ microcalorimeter (Microcal) under the same buffer conditions (20 mM Tris, pH 7.5, containing 5 mM NaCl), except for experiments using PP5, where we omitted EDTA and used 1 μM MnCl_2_. In experiments using nucleotide, we used 5 mM AMPPNP and 6 mM MgCl_2_. For experiments using Aha1 with AMPPNP the buffer contained 100 mM NaCl. Similarly, Sgt1 experiments also included 100 mM NaCl in the buffer. To assess the binding of LA1011 to Hsp90 a variety of Hsp90—co-chaperone complexes we generally injected with aliquots of 2 mM LA1011 (prepared as a 50 mM stock in the same buffer) into the microcell containing the protein complex at concentrations of 20 or 30 μM at 30 ^o^C. To assess co-chaperone binding to Hsp90 and Hsp90-LA1011 complex, we generally injected aliquots of co-chaperone at concentrations ranging from 200 to 400 μM into the microcell containing the Hsp90 or Hsp90-LA1011 complex at concentrations ranging from 20 to 30 μM of Hsp90 or Hsp90 in complex with 2 mM LA1011 at 30 ^o^C. Heats of dilution were determined by diluting the injectant into the buffer. Data were fitted using a curve-fitting algorithm (Microcal Origin) either as a one-site binding event or as two independent binding sites as required.

### SwissDock binding predictions for LA1011

Docking of LA1011 was performed on Autodock Vina 1.2.5 to estimate the free energy for the binding of LA1011 to both the Xtal and MD sites of the C-terminal domain of yeast Hsp90.^43^, ^44^ As targets, for Xtal-site docking we used the Hsp90 model from the crystal structure of the LA1011 complex with Hsp90 (PDB 8OXU), and for MD-site docking we used a yeast-model structure based on the human molecular-dynamics structure,^11^, ^12^ which was built using the i-Tasser server and assigning the molecular dynamics structure as a template.^45^, ^46^

## Results and conclusions

### Isothermal titration calorimetry curve fitting parameters

Our previous ITC experiments used a stoichiometry of binding of 2:1 representing a Hsp90 dimer binding a single molecule of LA1011. This assumed that LA1011 only bound to our Xtal site, but our molecular dynamics simulations have also suggested the presence of a second site (MD site). Ideally fittings parameters for ITC experiments should take this into account. However, in our experience, the fitting of two independent events with a weakly binding compound effectively returns *K*d values with large errors. To circumvent this limitation, we can alternatively fit the two sites as equal thermodynamic events, and the *K*d value or values(s) returned then act as an indicator for changes in the overall affinity for LA1011 binding. In fact, using AutoDock Vina predictions for binding affinities to each site, we find that the predicted free energy for LA1011 binding is not that dissimilar between the two sites. The free energy scores for LA1011 docking were −6.246 (approximates to a *K*d of 26 μM) for the Xtal site and −5.655 kcal mol^-1^ (approximates to a *K*d of 54 μM) for the MD site. This approach, ultimately, helps to answer our question of how do specific Hsp90—co-chaperone complexes affect LA1011 binding? Thus, our overall approach is to use a stoichiometry of 1:1 that represents the binding of two LA1011 molecules (because there are two binding sites) per Hsp90 dimer, except where we are confident that the stoichiometry is 2:1 (Hsp90 dimer and one LA1011 molecule). Examples of a 2:1 binding event would be where helix-7 of a TPR domain-containing protein would occupy the Xtal site so that only the MD site is available or in cases where the MD site might be unavailable and the Xtal site free. Thus, prior to choosing the ITC fitting parameters, we first consider the availability of both LA1011 binding sites for complexes of Hsp90 with its co-chaperone partner. However, in Table 1 we present both fits (2:1 and 1:1), since our data can be fitted accurately using either fit, and the affinities observed from each type of fit lead to the same overall conclusion for that interaction. Consequently, we will present in the main text of this manuscript the *K*d values representing a 1:1 fit, except where otherwise stated.

**Table 1:** The effect of Hsp90—co-chaperone complex on the binding of LA1011.

Hsp90—co-chaperone complex	Binding partner (2 mM LA1011)	*K*d (μM) (2:1)	*K*d (μM) (1:1)
Hsp90	LA1011	28.9 ± 1.7	19.8 ± 2.2
Hsp90-AMPPNP	LA1011	73.0 ± 3.1	58.8 ± 24.2
Hsp90-FKBP51	LA1011	79.4 ± 33.5	NA
Hsp90-FKBP52	LA1011	56.2 ± 5.8	NA
Hsp90-CHIP	LA1011	69.0 ± 2.0	56.2 ± 26.2
Hsp90-p23-PNP	LA1011	73.0 ± 4.5	58.8 ± 3.5
Hsp90-Aha1	LA1011	53.2 ± 5.5	117.5 ± 8.0
Hsp90-HOP	LA1011	8.8 ± 1.3	8.8 ± 1.3
Hsp90-CDC37	LA1011	9.5 ± 2.5	4.2 ± 0.8
Hsp90-Sgt1	LA1011	8.2 ± 1.0	NA
Hsp90-Aha1-PNP	LA1011	NB	NB
Hsp90-Hch1-PNP	LA1011	NB	NB
Hsp90-PP5	LA1011	NB	NB

Relative to the Hsp90 control (*Kd = 20.4 μM), the binding of LA1011 is affected in one of three ways. Either the binding is weakened, strengthened, or LA1011 fails to bind (NB). NA = not applicable.*

### Co-chaperones that bind nucleotide-free Hsp90 influence the binding of LA1011

**Fig. 2:**
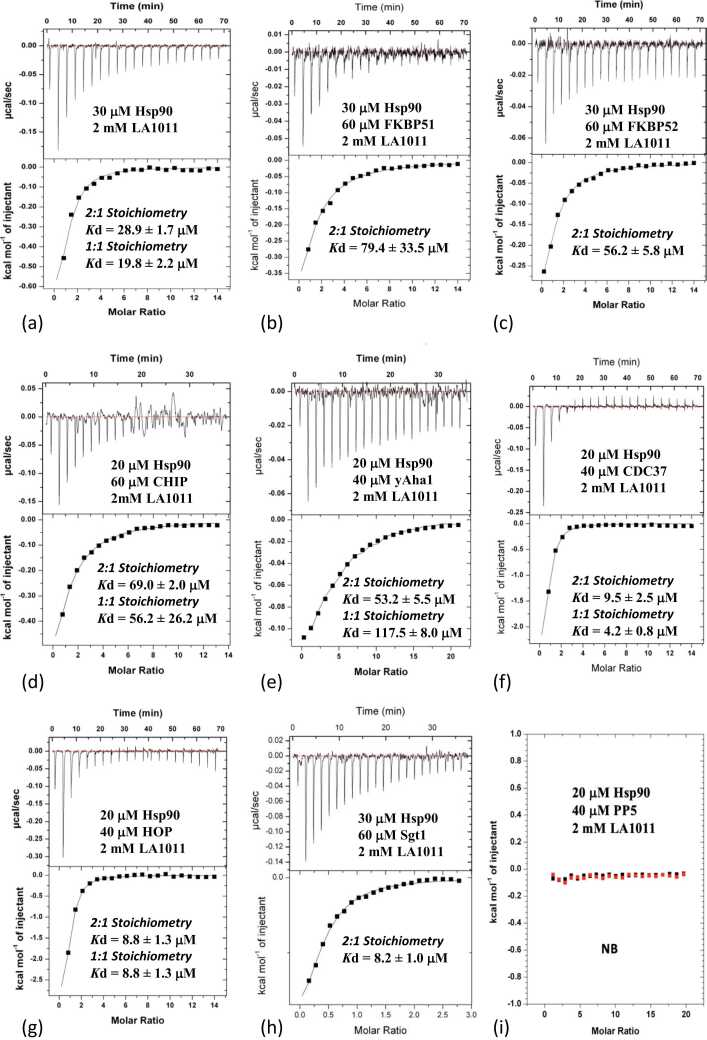
ITC experiments evaluating the binding of LA1011 to a variety of Hsp90—co-chaperone complexes. The concentrations and interacting proteins are shown in each panel, as are estimates of the *K*d values for each interaction. (a) LA1011 binding to Hsp90. (b) LA1011 binding to the Hsp90-FKBP51 complex. (c) LA1011 binding to the Hsp90-FKBP52 complex. (d) LA1011 binding to the Hsp90-mouse CHIP complex. (e) LA1011 binding to the Hsp90-yeast Aha1 complex. (f) LA1011 binding to the Hsp90-CDC37 complex. (g) LA1011 binding to the Hsp90-HOP complex. (h) LA1011 binding to the Hsp90-Sgt1 complex and (i) LA1011 binding to the Hsp90-PP5 complex.

To investigate the influence of co-chaperones on LA1011 binding with nucleotide-free Hsp90, we used ITC. Previously we had fitted the binding of LA1011 using the assumption of a single binding site being present on Hsp90, and here we find using the same fit for the current experiment a *K*d of 28.9 ± 1.7 μM (Figure 2(a) and Table 1) for the interaction, which was consistent with our previous study.^12^ Furthermore, when assuming two LA1011 molecules are binding a Hsp90 dimer, we obtained a similar affinity (*K*d = 19.8 ± 2.2 μM). For the Hsp90-FKBP51 complex, the Xtal site is blocked by helix-7 of the TPR domain of FKBP51; hence, the best fit is a 2:1 (Hsp90:LA1011) stoichiometric fit. We found that the *K*d for LA1011 binding was 79.4 ± 33.5 μM (Figure 2(b)), suggesting weaker binding to the Hsp90-FKBP51 complex than for Hsp90 alone (*K*d = 28.9 ± 1.7 μM; Figure 2(a)). The weakened binding is probably due to the inaccessibility of the Xtal site (bound by helix-7 of the TPR domain of FKBP51), and the interaction reflects residual binding to the MD site. This was in line with the predicted binding using AutoDock Vina, which estimated binding affinity at the Xtal site to have a *K*d of 26 μM and *K*d of 54 μM for the MD site. Similarly, the Hsp90 complex containing FKBP52 returned a *K*d of 56.2 ± 5.8 μM (Figure 2(c)), representing a weaker affinity than the Hsp90 control (*K*d of 28.9 ± 1.7 μM; Figure 2(a)). For mouse CHIP there is no evidence that its TPR domain interacts with the Xtal site, and consequently we used a 1:1 stoichiometric fit (Hsp90:LA1011). We found that the overall affinity for LA1011 was weakened (*K*d of 56.2 ± 26.2; Figure 2(d)) relative to Hsp90 alone (*K*d = 28.9 ± 1.7 μM; Figure 2(a)). Similarly, 1:1 stoichiometric fit for the nucleotide-free Hsp90-Aha1 complex returned a reduced affinity for LA1011 (*K*d = 117.5 ± 8.0 μM [1:1]; Figure 2(e)). In contrast, the Hsp90-CDC37, Hsp90-HOP, and Hsp90-Sgt1 complexes appear to increase the affinity for LA1011. The Hsp90-CDC37 complex displayed a *K*d of 4.2 ± 0.8 μM ([1:1]; Figure 2(f)), the Hsp90-HOP complex returned a *K*d of 8.8 ± 1.3 μM ([1:1]; Figure 2(g)) and the Hsp90-Sgt1 complex returned a *K*d of 8.2 ± 1.0 μM ([2:1]; Figure 2(h)). For Sgt1, we could only fit binding using a 2:1 (Hsp90:LA1011) stoichiometry. However, we were particularly surprised to find that the Hsp90-PP5 complex completely blocked LA1011 binding (Figure 2(i)). PP5 is known to bind the Xtal site using its conserved ϕ/Yxxϕϕ motif found in helix-7 of its TPR domain, but the results suggest that the MD site is also inaccessible.

In conclusion, our results suggest that the co-chaperones enforce a variety of conformations on Hsp90 that either increase, weaken, or block the interaction of LA1011 with Hsp90. In particular, we see that the co-chaperones CDC37, HOP, and Sgt1, which enforce an open Hsp90 conformation, often as part of the early complex of the chaperon cycle, increase the affinity of Hsp90 for LA1011.^47^, ^48^, ^49^ In contrast, the Hsp90 intermediate-cycle co-chaperones FKBP51 and FKBP52 showed reduced affinity for LA1011 and the weakened interaction seen most likely represents residual binding of LA1011 to the MD site. The Hsp90-dependent E3 ubiquitin ligase CHIP and the late-cycle co-chaperone Aha1 (bound to nucleotide-free Hsp90), where also found to reduce the binding of LA1011, but the mechanisms for these effects remain unknown.

### The nucleotide-loaded form of Hsp90 and co-chaperones that promote its closed conformation weaken the LA1011 interaction

**Fig. 3:**
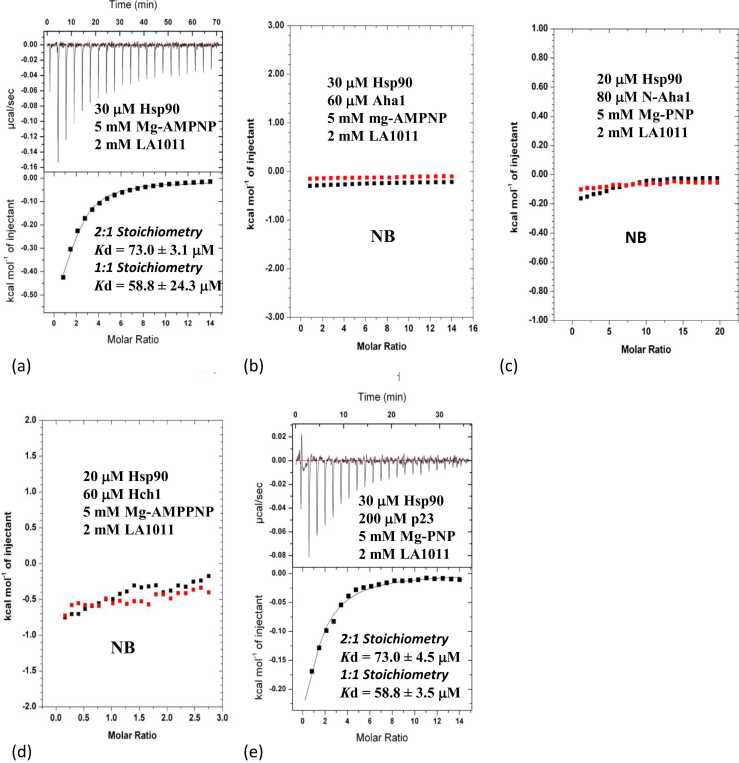
ITC experiments evaluating the binding of LA1011 to a variety of nucleotide-bound Hsp90—co-chaperone complexes. The concentrations and interacting proteins are shown in each panel as are estimates of the *K*d values for each interaction. (a) LA1011 binding to the AMPPNP-Hsp90 complex. (b) LA1011 binding to AMPPNP-Hsp90-yeast Aha1 complex. (c) LA1011 binding to AMPPNP-Hsp90—N-terminal domain yeast Aha1 complex. (d) LA1011 binding to the AMPPNP-Hsp90-Hch1 complex and (e) LA1011 binding to the AMPPNP-Hsp90-p23 complex.

Next, we evaluated the binding of LA1011 with nucleotide-loaded Hsp90 and found that its binding affinity was weaker (58.8 ± 24.2 μM; [1:1]; Figure 3(a)) relative to nucleotide-free Hsp90 (*K*d = 19.8 ± 2.2 μM; [1:1]; Figure 2(a)). This suggests that the closed conformation of Hsp90 disfavors LA1011 binding relative to an open complex of Hsp90 (Hsp90-CDC37, Hsp90-HOP, and Hsp90-Sgt1; Figure 2(f)-(h)). Furthermore, the binding of LA1011 was completely disrupted when using the AMPPNP-Hsp90-yeast Aha1 complex (Figure 3(b)), which represents a fully closed and ATPase-competent form of Hsp90. This suggests that Aha1 promotes a closed conformation where both LA1011 sites (Xtal and MD) are essentially inaccessible to LA1011. Similarly, LA1011 failed to bind nucleotide-bound Hsp90 in complex with the N-terminal domain of Aha1 and the homologous Hch1 protein (Figure 3(c) and (d)), suggesting that the N-terminal domain of Aha1 is wholly sufficient for the inhibitory effect of Aha1 on LA1011 binding. In contrast to Aha1, the nucleotide-bound Hsp90-p23 complex, which also represents a closed conformation of Hsp90, did not block LA1011 binding (*K*d = 73. ± 4.5 μM; [1:1]; Figure 3(e)), but it was reduced relative to Hsp90 alone (*K*d = 28.9 ± 1.7 μM; [1:1]; Figure 2(a)). In conclusion, our results suggest that the closed AMPPNP-Hsp90-p23 complex is conformationally different in detail to that driven by Aha1 and that it disfavors LA1011 binding relative to the open conformation.

### Co-chaperone binding to the Hsp90-LA1011 complex

**Fig. 4:**
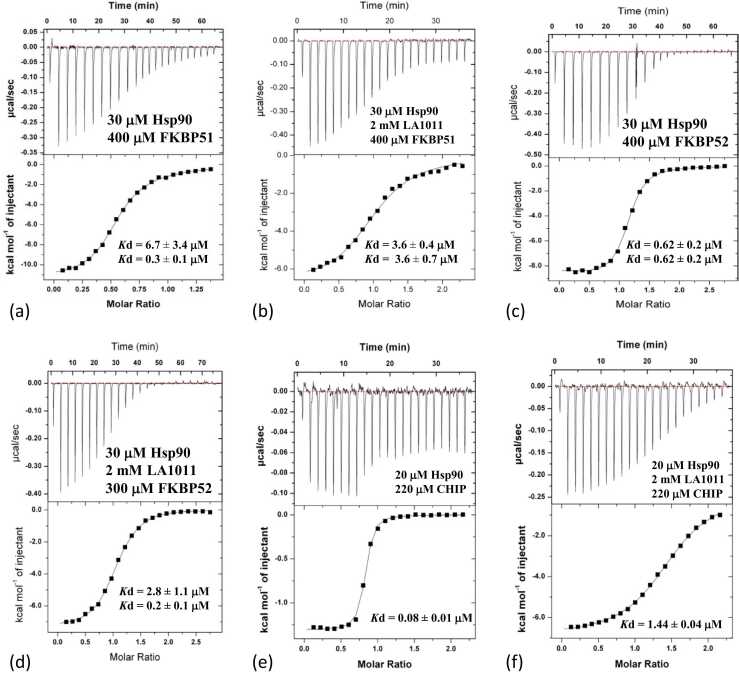
ITC experiments evaluating the binding of a variety of co-chaperones to a Hsp90 and Hsp90-LA1011 complex. The concentrations and interacting proteins are shown in each panel, as are estimates of the *K*d values for each interaction. Where co-chaperones potentially have two modes of binding, a two-site fit is used, which returns two *K*d values. (a) FKBP51 binding to Hsp90. (b) FKBP51 binding the Hsp90-LA1011 complex. (c) FKBP52 binding to Hsp90. (d) FKBP52 binding to the Hsp90-LA1011 complex. (e) CHIP binding to Hsp90 and (f) CHIP binding to the Hsp90-LA1011 complex.

**Fig. 5:**
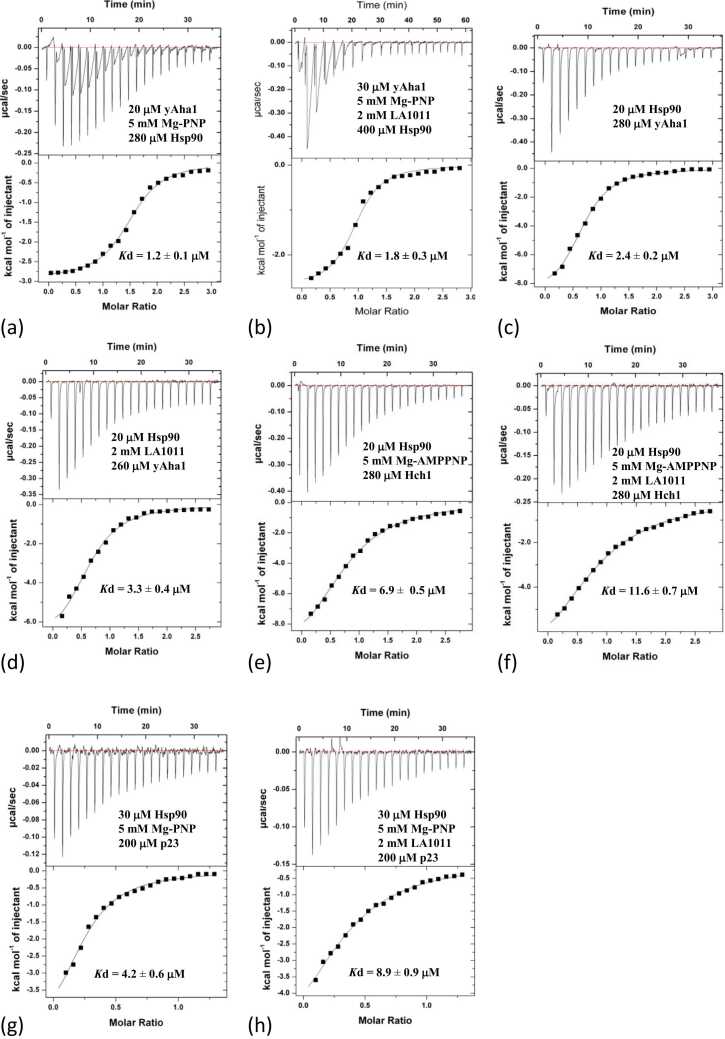
ITC experiments evaluating the binding of a variety of co-chaperones to Hsp90, AMPPNP-Hsp90, and the AMPPNP-Hsp90-LA1011 complex. The concentrations and interacting proteins are shown in each panel, as are estimates of the *K*d values for each interaction. Where co-chaperones potentially have two modes of binding, a two-site fit is used, which returns two *K*d values. (a) Hsp90-AMPPNP binding to yeast Aha1. (b) Hsp90-AMPPNP-LA1011 binding to yeast Aha1. (c) Yeast Aha1 binding to Hsp90. (d) Yeast-Aha1 binding to the Hsp90-LA1011 complex. (e) Hch1 binding to the AMPPNP-Hsp90 complex. (f) Hch1 binding to the AMPPNP-Hsp90-LA1011 complex. (g) p23 binding to the AMPPNP-Hsp90 complex and (h) p23 binding to the AMPPNP-Hsp90-LA1011 complex.

**Fig. 6:**
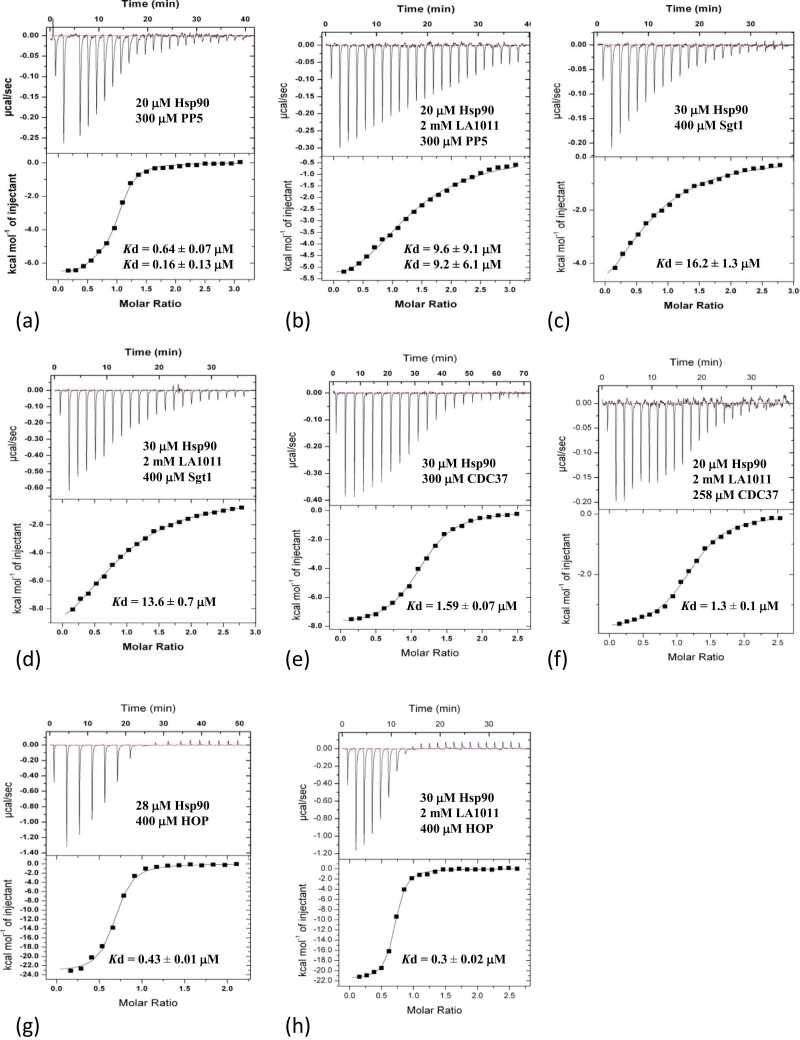
ITC experiments evaluating the binding of a variety co-chaperones that bind an open-conformation of HSP90 in the absence and presence of LA1011. The concentrations and interacting proteins are shown in each panel, as are estimates of the *K*d values for each interaction. Where co-chaperones potentially have two modes of binding, a two-site fit is used, which returns two *K*d values. (a) PP5 binding to Hsp90. (b) PP5 binding to the Hsp90-LA1011 complex. (c) Sgt1 binding to Hsp90. (d) Sgt1 binding to the Hsp90-LA1011 complex. (e) CDC37 binding to Hsp90. (f) CDC37 binding to the Hsp90-LA1011 complex. (g) HOP binding to Hsp90 and (h) HOP binding to the Hsp90-LA1011 complex.

Although we had evaluated the binding of LA1011 to numerous Hsp90—co-chaperone complexes, we wondered whether the Hsp90-LA1011 complex could significantly influence the interaction of co-chaperones with Hsp90. The binding of FKBP51 was fitted as a two-site binding event, where one molecule of FKBP51 binds one conserved MEEVD motif of Hsp90 and another FKBP51 molecule binds a single conserved MEEVD motif and simultaneously uses helix-7 of its TPR domain to interact with the Xtal site on Hsp90. After fitting the data, we observed a pair of *K*d values of 0.3 ± 0.1 and 6.7 ± 3.4 μM (Figure 4(a) and Table 2). However, when we fitted data from an experiment using a Hsp90-LA1011 complex, we observed the loss of the higher-affinity binding event (both sites fitting with a *K*d of 3.6 μM). Loss of affinity was particularly evident from the reduction of enthalpy observed (Figure 4(a) and (b)). Similar results were obtained with FKBP52. FKBP52 bound free Hsp90 with two *K*d values both approximating to 0.62 μM (Figure 4(c)) and with the Hsp90-LA1011 complex, we saw one of the *K*d values significantly increase (*K*d = 2.8 ± 1.1 μM), while the other approximated to a *K*d of 0.2 ± 0.1 μM, which is similar to the previous value of 0.62 μM (Figure 4(d)). The results suggest that LA1011 compromises the overall interaction of FKBP51 and FKBP52 with Hsp90, which most likely arises because LA1011 can occupy the Xtal site required for high-affinity binding by these co-chaperones. Next, we investigated the binding of CHIP, which is dimeric in nature,^38^ and found it interacted with Hsp90 with a *K*d of 0.08 ± 0.01 μM (Figure 4(e)). However, the affinity of mouse CHIP for Hsp90 decreased in the presence of LA1011 (*K*d of 1.44 ± 0.04 μM; Figure 4(f)). For Aha1 binding to the Hsp90-AMPPNP-LA1011 complex, we saw a slight reduction in affinity (Kd = 1.8 ± 0.3 µM) relative to the control complex lacking LA1011 (Kd = 1.2 ± 0.1 µM; Figure 5(a) and (b)). Interestingly, in the absence of AMPPNP, the interaction between Aha1 and Hsp90 was weaker for both Hsp90 alone (*K*d = 2.4 ± 0.2 μM) and the Hsp90-LA1011 complex (*K*d = 3.3 ± 0.4 μM; Figure 5(c) and (d)) and there was perhaps a slight preference for LA1011-free Hsp90. A similar effect was seen in Hch1 experiments with LA1011-free Hsp90 (*K*d = 6.9 ± 0.5 μM; Figure 5(e)) and LA1011-bound Hsp90 (*K*d = 11.6 ± 0.7 μM; Figure 5(f)). This pattern repeated itself using p23, where the Hsp90-AMPPNP-LA1011 complex also had a small negative effect on p23 binding (*K*d = 4.2 ± 0.6 μM for Hsp90-AMPPNP complex and a *K*d of 8.9 ± 0.9 μM for the Hsp90-AMPPNP-LA1011 complex; Figure 5(g) and (h)). For the PP5 interaction with Hsp90, we found that PP5 bound Hsp90 with high affinity (*K*d = 0.16 ± 0.13 and *K*d = 0.64 ± 0.07 μM; Figure 6(a)), but its binding to the Hsp90-LA1011 complex was compromised (*K*d = 9.6 ± 9.1 and *K*d = 9.2 ± 6.1 μM; Figure 6(b)). PP5 is known to interact with the Xtal site by using its conserved ϕ/Yxxϕϕ motif in helix-7 of its TPR domain.^23^, ^50^ Finally, the Hsp90-LA1011 complex did not significantly affect the binding of Sgt1, CDC37, and HOP (Figure 6(c)-(h)). These co-chaperones tend to be part of early complexes in the Hsp90 cycle and we noticed that for these co-chaperones, their binding in the presence of Hsp90-LA1011 complex was consistently and marginally stronger than for binding to Hsp90 alone. However, we cannot at this stage say whether such small differences are of any biological significance. The dissociation constants we obtained were a *K*d of 16.2 ± 1.3 μM (Figure 6(c)) for Sgt1 binding to Hsp90 and a *K*d of 13.6 ± 0.7 μM (Figure 6(d)) for binding to Hsp90-LA1011 complex. For CDC37, we obtained a *K*d of 1.59 ± 0.7 μM for binding to Hsp90 (Figure 6(e)) and *K*d of 1.3 ± 0.1 μM (Figure 6(f)) for binding to the Hsp90-LA1011 complex, and finally for HOP, we obtained a *K*d of 0.43 ± 0.1 μM (Figure 6(g)) for binding to Hsp90 and a *K*d of 0.3 ± 0.02 μM (Figure 6(h)) for binding to the Hsp90-LA1011 complex. As these differences are small, it is difficult to draw any concrete conclusion, but increased binding affinity for these co-chaperones may be due to LA1011 favoring an open state to which these early-stage co-chaperones prefer to bind to.

**Table 2:** The effect of the Hsp90-LA1011 complex on the binding of Hsp90 co-chaperones.

ITC cell component	Binding partner (injectant)	*K*d (μM)
Hsp90	FKBP51	6.7 0.3
Hsp90-LA1011	FKBP51	3.6 3.6
Hsp90	FKBP52	0.54 0.54
Hsp90-LA1011	FKBP52	2.8 0.2
Aha1	Hsp90-PNP	1.2
Aha1	Hsp90-LA1011-PNP	1.8
Hsp90	Hch1	6.9
Hsp90-LA1011	Hch1	11.6
Hsp90	CHIP	0.08
Hsp90-LA1011	CHIP	1.44
Hsp90	HOP	0.43
Hsp90-LA1011	HOP	0.3
Hsp90	CDC37	1.59
Hsp90-LA1011	CDC37	1.3
Hsp90-PNP	p23	4.2
Hsp90-LA1011-PNP	p23	8.9
Hsp90	PP5	0.64 0.16
Hsp90-LA1011	PP5	9.6 9.2
Hsp90	Sgt1	16.2
Hsp90-LA1011	Sgt1	13.6

For co-chaperones where one molecule binds the MEEVD motif and another binds a second MEEVD motif together with the helix-7 binding site, a two-site fit is used, which returns two *Kd values.*

### The effect of LA1011 on the co-chaperone directed regulation Hsp90 ATPase activity

**Fig. 7:**
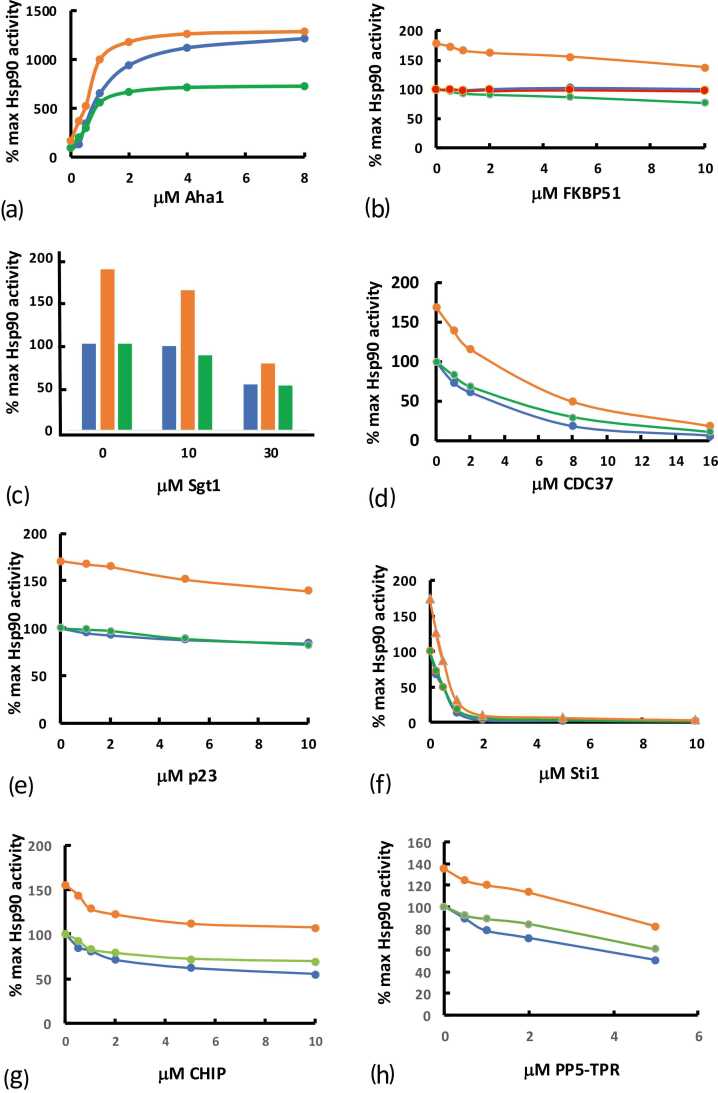
The effect of co-chaperones on the LA1011-stimulated ATPase activity of Hsp90. Blue curves represent the control assays where the co-chaperone effect on the Hsp90 ATPase activity is evaluated. The orange cures are assays conducted with LA1011-stimulated Hsp90 ATPase activity. The green curves are the normalized data from the LA1011-stimulated Hsp90 assays. Where the normalized data (green curve) fails to coincide with the control data (blue line), this indicates an LA1011 effect on co-chaperone action. Assays in the presence of (a) yeast Aha1, (b) FKBP51, (c) Sgt1, (d) CDC37, (e) p23, (f) Sti1, (g) mouse CHIP, and (h) PP5.

To assess the effect of LA1011 on the mechanistic action of co-chaperones, we used the lactate-dehydrogenase and pyruvate-kinase-linked Hsp90 ATPase assay. We first assessed the effect of each co-chaperone on Hsp90 ATPase activity and then compared its effect on a Hsp90-LA1011 complex. The Hsp90-LA1011 data were then normalized, and any deviation from the control data (Hsp90—co-chaperone assay lacking LA101) after normalization was an indication that LA1011 had an effect on co-chaperone action (Figure 7). It appears that in the presence of LA1011, significantly less Aha1 is required to fully activate Hsp90. This is perhaps not surprising, since LA1011 and Aha1 are both activators of Hsp90 ATPase activity (Figure 7(a) and Supplementary Figure 1, shows the statistical analysis). For FKBP51 we saw inhibition of LA1011-stimulated Hsp90 ATPase activity (Figure 7(b)), which is an indication of the competitive binding between FKBP51 and LA1011 for Hsp90. In contrast, Sgt1, CDC37, p23, and Sti1 (the yeast homolog of human HOP) were unaffected by the presence of LA1011 in the ATPase assay (Figure 7(c)-(f)). For mouse CHIP, we report that it is an inhibitor of the ATPase activity of Hsp90 and find that LA1011 affects CHIP’s ability to inhibit Hsp90 ATPase activity (Figure 7(g) and Supplementary Figure 2). Finally, we assayed the effect of the TPR domain of PP5 on the LA1011-stimulated activity of Hsp90. We found that LA1011 had a small but significant effect on the ability of the TPR domain of PP5 to inhibit the ATPase activity of Hsp90 at sub-stoichiometric molar ratios of the TPR-PP5 domain to Hsp90 when using 2 μM of Hsp90 (Figure 7(h)).

## Discussion

**Fig. 8:**
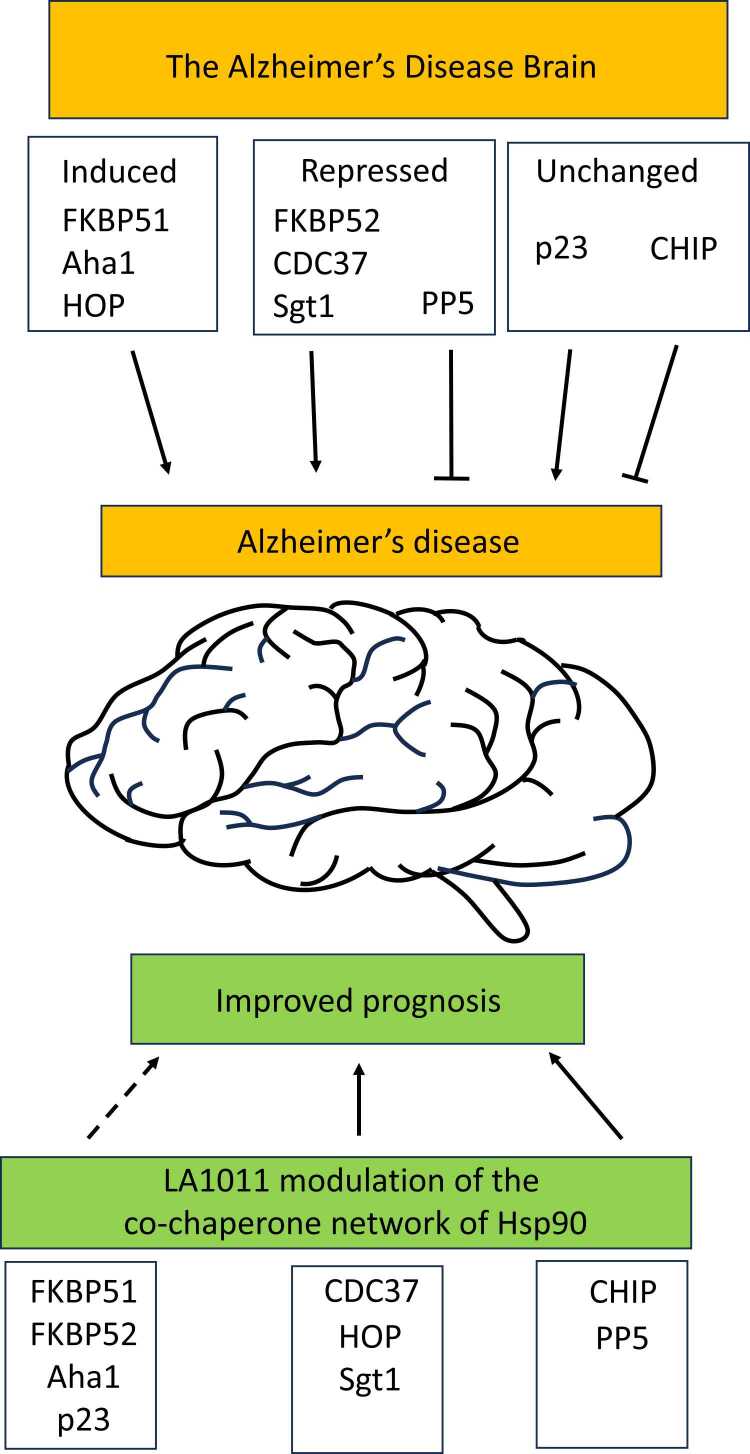
LA1011 modulation of the co-chaperone network of an Alzheimer’s disease brain. AD changes in the expression of various co-chaperones are shown above the image of the brain. Below the image of the brain, we show how the co-chaperones are modulated by LA1011. Above the brain, solid arrows indicate that co-chaperones have been associated with the development of AD. HOP has been identified in the “induced” cluster in both the aged and the AD brain as part of the TPR-domain co-chaperone group.^18^ T-shaped arrows indicate co-chaperones that help to limit disease progression. Below the brain, dashed arrows represent modulated co-chaperone action, where we assume this restricts development of AD, whereas solid arrows indicate co-chaperones that may promote AD. However, their overall action on AD development might now be modified due to other changes in the chaperone network brought about by LA1011 treatment.

Age-related changes in the chaperone network can lead to imbalances in the regulation of the Hsp90 chaperone system, which may ultimately promote disease-associated processes^18^, ^19^, ^21^, ^30^, ^51^, ^52^, ^53^ that may consequently confer further changes on the chaperone network. Differences in age-related changes relative to those in the AD brain can help identify specific co-chaperones that are involved in disease-promoting processes. Co-chaperones reported as supporting the development of AD include CDC37, p23, Aha1, FKBP52, and FKBP51, while co-chaperones such as CHIP and PP5 may help limit disease progression, a topic that has recently been reviewed.^21^, ^54^, ^55^ However, understanding how to modulate an imbalanced network to prevent the development of disease is not only difficult but it is a critical and an essential starting point for the successful development of drugs that can improve the prognosis of AD. A major development towards new drugs against AD is the identification of two Hsp90-binding sites for LA1011.^11^, ^12^ In this study, we show that LA1011 can modulate the mechanistic action of co-chaperones on Hsp90, and the overall modulatory effects of LA1011 are summarized in Figure 8.

**Fig. 9:**
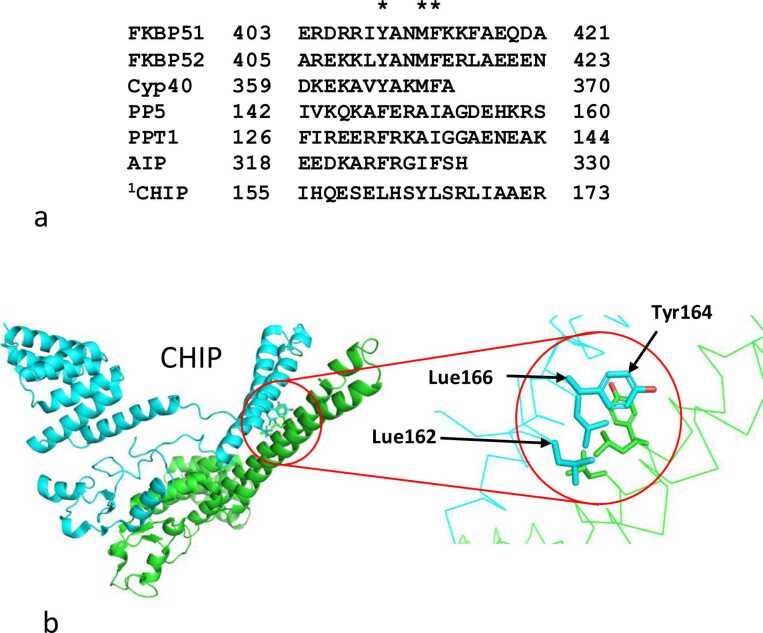
An alignment of a variety of amino acid sequences from helix-7 of various TPR domain-containing co-chaperones. (a) Alignment of amino acid sequences that possess an apparent conserved motif, consisting of three conserved (yellow highlight) hydrophobic residues (ϕ/Yxxϕϕ, where ϕ represents a hydrophobic residue and position one may also be tyrosine (Y)). ^1^Unlike other TPR domains, the motif found in CHIP actually forms the dimer interface of CHIP. (b) The dimer interface of mouse CHIP showing that the apparent motif identified in helix-7 of CHIP may not itself be available for binding to the Xtal site of Hsp90.

We find that three TPR domain-containing proteins, FKBP51, FKBP52, and mouse CHIP weaken the binding of LA1011. We suspect this weakened binding is a reflection of LA1011 binding to the MD site, since the weak LA1011-Hsp90 interaction affinity (*K*d = 28.9 μM) would not be sufficient to displace the tighter Hsp90—co-chaperone interactions, which display micromolar affinities (*K*d = 0.3 μM, FKBP51; 0.62, μM FKBP52 and 0.08 μM CHIP). However, direct evidence for residual binding to the MD site is not presently available. Furthermore, although CHIP possesses a hydrophobic motif in helix-7 of its TPR domain, similar to the conserved ϕ/Yxxϕϕ motif known to interact with the Xtal site, CHIP’s motif actually forms part of the dimer interface in mouse CHIP^38^ (Figure 9). Whether CHIP rearranges to use this motif to bind the Xtal site of Hsp90 or whether it influences the binding of LA1011 by another mechanism is currently unknown. In contrast, it was clear that HOP (Sti1) does not appear to have a helix-7 binding motif, and this is consistent with structural studies that show HOP does not interact with the Xtal site of Hsp90.^48^ Instead, we find that HOP approximately doubles the affinity of LA1011 for Hsp90. HOP is a co-chaperone found in early complexes of the chaperone cycle, and as with other co-chaperones of early complex assembly, like CDC37 and Sgt1, we also found these approximately double the affinity for LA1011. Another observation that requires further investigation is that for PP5, which binds the Xtal site of Hsp90 in a similar way to that of FKBP51, but nonetheless prevents LA1011 binding altogether. It, therefore, appears that PP5 is probably influencing the MD site for LA1011 binding, which distinguishes it from the other TPR domain proteins such as FKBP51 and FKBP52. Clearly, there is a fundamentally different effect on Hsp90 conformation imposed by PP5 compared to FKBP51 and FKBP52.

For nucleotide and co-chaperone driven closed conformations of Hsp90, we found that the Hsp90 affinity for LA1011 was weakened to different degrees. AMPPNP and AMPPNP-p23 driven conformations had a similar affinity for LA1011, whereas the AMPPNP-Aha1 conformation blocked LA1011 binding altogether. Therefore, it would appear that LA1011 and Aha1 binding to Hsp90 are mutually exclusive events and that as with other allosteric activators, such as compound 18 and 19,^15^ LA1011 reduces the requirement for Aha1 for maximum stimulation of Hsp90 ATPase activity. Hence, it appears that LA1011 may set up structural changes in Hsp90 such that the overall requirement for Aha1 for maximum stimulation of Hsp90 ATPase activity is reduced. This appears to be evident in our ATPase activities, where saturation of ATPase activity is achieved with a lower stoichiometric molar ratio of Aha1 to Hsp90 (Figure 7(a)).

Collectively, our results show that co-chaperones promote multiple conformations of Hsp90, each differing in specific detail, which ultimately affects the binding of LA1011 in different ways, and conversely, LA1011 affects the regulation of Hsp90 by affecting the binding of such co-chaperones with Hsp90. This is perhaps not surprising for co-chaperones, which favor specific conformations of Hsp90. While some of the effects on co-chaperone function could be perceived to be beneficial to the prognosis of AD, such as reducing the Hsp90-dependent activity of FKBP51, other effects on CHIP and PP5 activity might appear to be counterproductive. Inhibition of PP5 and CHIP activity, for example, would be considered undesirable, since CHIP is an E3 ubiquitin ligase responsible for clearance of stalled Hsp90-client protein complexes and PP5 can dephosphorylate tau. However, in the AD mouse model, this does not appear to have been a limiting factor that would prevent an improvement in the prognosis of AD. Whether this is also true for AD patients remains to be seen, but nonetheless, for now, this remains a slight concern. It may, however, be noteworthy to mention that PP5 and CHIP show some of the strongest binding affinity to Hsp90 amongst the co-chaperones we have investigated, and consequently their activity could be less influenced by LA1011. In fact, we saw that PP5 could prevent LA1011 binding altogether. Furthermore, CHIP expression remains unchanged in the AD brain, while PP5 expression is repressed.^19^ Thus, disruption of their activity by LA1011 may, in response, potentially increase their expression. In contrast, FKBP51 has less potential for upregulation, as it is already substantially induced in the AD brain. The ability of LA1011 to activate Hsp90, in a similar way to that seen for Aha1, is also a slight concern, since Aha1 is known to promote AD^56^, ^57^ and, in fact, Aha1 levels in the AD brain are induced above those of an age-related level.^18^, ^19^ However, it is important not to oversimplify the effects on specific co-chaperones of Hsp90 and the development of AD. The negative effects of Aha1 could be negated due to other changes in the chaperone network in response to LA1011 administration. Clearly, further investigation is needed to reveal the consequences of modulating specific co-chaperones on the overall co-chaperone network. However, for p23 and CDC37, it has been reported that they can promote AD processes. It appears from our experiments that LA1011 can reduce the interaction of p23 with Hsp90 (Figure 5(g) and (h)) although this is marginal, at best. For CDC37 there is a slight increase in affinity for Hsp90, but the biological significance of this is not easy to assess. However, while understanding the effects of LA1011 on co-chaperone function is invaluable, it is also essential that we understand the effect of LA1011 on Hsp90-dependent client protein binding and activation. For example, it is known that C-terminal binding compounds of Hsp90 can affect the binding of the model client protein delta131delta (Δ131Δ. Thus, there is a potential for LA1011 to reduce tau interaction with Hsp90, thus negating some of the disease-promoting effects that might be imposed by LA1011 activation of Hsp90 or its negative effects on CHIP and PP5 activity. Clearly, this study raises many questions, which we are now actively investigating.

In conclusion, we found multiple co-chaperone systems are modulated by the LA1011 interaction with Hsp90. Specifically, inhibition of FKBP51-Hsp90 and Aha1-Hsp90 activity, which promote AD, are promising modulatory changes. In contrast, inhibition of FKBP52-Hsp90, CHIP-Hsp90, and PP5-Hsp90 activities, at first sight, appears to be unfavorable. However, this is a simplistic view, and modulation of a single Hsp90—co-chaperone activity, such as the Hsp90-FKBP51 complex, may be sufficient to overcome negative effects from the modulation of other Hsp90 complexes. Consequently, these widespread effects on Hsp90 function warrant a close examination of the downstream effects on tau phosphorylation in the context of an AD brain and whether changes in regulation are beneficial or disadvantageous in treating AD. However, it is promising that changes in the mechanistic action of the Hsp90—co-chaperone network, at least in the AD mouse model appears to improve the prognosis of AD. Whether this now translates to AD patients in the clinic remains to be seen, but nonetheless, we now have a much better understanding on the mechanistic effects of LA1011 on the Hsp90—co-chaperone network.

## CRediT authorship contribution statement

**Xavier Jeanne:** Writing – review & editing, Methodology, Formal analysis. **Jasmeen Oberoi:** Writing – review & editing, Methodology. **Mark S. Roe:** Writing – review & editing, Funding acquisition. **Matthias Baud:** Writing – review & editing, Funding acquisition. **John Spencer:** Writing – review & editing, Funding acquisition. **Zsolt Torok:** Writing – review & editing, Funding acquisition. **Laszlo Vigh:** Writing – review & editing, Funding acquisition. **Chrisostomos Prodromou:** Writing – review & editing, Writing – original draft, Validation, Supervision, Project administration, Methodology, Investigation, Funding acquisition, Formal analysis, Conceptualization.

## Declaration of generative AI in scientific writing

No significant use of generative AI was used.

## Declarations of interest

X.J., J.O., M.S.R., M.B., J.S., and C.P. declare no conflicts of interest. L.V. and Z.T. declare themselves named on the Richter Gedeon Plc. pharmaceutical company patent number 10660789 (https://patents.justia.com/patent/10660789).

## Data Availability

Raw data for experiments can be found at https://doi.org/10.25377/sussex.30576458.

## Appendix A Supplementary material

Supplementary Material Figure 1. Paired sample *T*-test statistical analysis of ATPase assays. Statistical analysis for (a) Aha1; (b) FKBP51; (c) Sti1; (d) Sgt1 1–280; (e) CDC37; (f) p23; (g) CHIP; and (h) PP5. Confidence levels are indicated by one or more asterixis. (*) *P* value < 0.5, significant; (**) *P* value < 0.01, very significant and (***) *P* value < 0.001, highly significant

## References

[1] Prodromou C., Bjorklund D.M. (2022). Advances towards understanding the mechanism of action of the Hsp90 complex. Biomolecules.

[2] Prodromou C. (2016). Mechanisms of Hsp90 regulation. Biochem J.

[3] Prodromou C. (2012). The “active life” of Hsp90 complexes. Biochim Biophys Acta.

[4] Roe S.M., Prodromou C., O.Brien R., Ladbury J.E., Piper P.W., Pearl L.H. (1999). The structural basis for inhibition of the Hsp90 molecular chaperone, by the anti-tumour antibiotics radicicol and geldanamycin. J Med Chem.

[5] Pearl L.H., Prodromou C., Workman P. (2008). The Hsp90 molecular chaperone: an open and shut case for treatment. Biochem J.

[6] Doi T., Yamamoto N., Ohkubo S. (2024). Pimitespib for the treatment of advanced gastrointestinal stromal tumors and other tumors. Future Oncol.

[7] D'Annessa I., Sattin S., Tao J. (2017). Design of allosteric stimulators of the Hsp90 ATPase as new anticancer leads. Chemistry.

[8] Cassiano C., Morretta E., Costantini M. (2021). Analysis of Hsp90 allosteric modulators interactome reveals a potential dual action mode involving mitochondrial MDH2. Bioorg Chem.

[9] Ferraro M., D'Annessa I., Moroni E. (2019). Allosteric modulators of HSP90 and HSP70: dynamics meets function through structure-based drug design. J Med Chem.

[10] D'Annessa I., Raniolo S., Limongelli V., Di Marino D., Colombo G. (2019). Ligand binding, unbinding, and allosteric effects: deciphering small-molecule modulation of HSP90. J Chem Theory Comput.

[11] Roe S.M., Torok Z., McGown A. (2023). The crystal structure of the Hsp90-LA1011 complex and the mechanism by which LA1011 may improve the prognosis of Alzheimer's disease. Biomolecules.

[12] Roe M.S., Wahab B., Torok Z., Horvath I., Vigh L., Prodromou C. (2018). Dihydropyridines allosterically modulate Hsp90 providing a novel mechanism for heat shock protein co-induction and neuroprotection. Front Mol Biosci.

[13] Bassanini I., D'Annessa I., Costa M., Monti D., Colombo G., Riva S. (2018). Chemo-enzymatic synthesis of (*E*)-2,3-diaryl-5-styryl-trans-2,3-dihydrobenzofuran-based scaffolds and their in vitro and in silico evaluation as a novel sub-family of potential allosteric modulators of the 90 kDa heat shock protein (Hsp90). Org Biomol Chem.

[14] Morelli L., Bernardi A., Sattin S. (2014). Synthesis of potential allosteric modulators of Hsp90 by chemical glycosylation of Eupomatenoid-6. Carbohydr Res.

[15] Sattin S., Tao J., Vettoretti G. (2015). Activation of Hsp90 enzymatic activity and conformational dynamics through rationally designed allosteric ligands. Chemistry.

[16] Vettoretti G., Moroni E., Sattin S. (2016). Molecular dynamics simulations reveal the mechanisms of allosteric activation of Hsp90 by designed ligands. Sci Rep.

[17] Lavery L.A., Partridge J.R., Ramelot T.A., Elnatan D., Kennedy M.A., Agard D.A. (2014). Structural asymmetry in the closed state of mitochondrial Hsp90 (TRAP1) supports a two-step ATP hydrolysis mechanism. Mol Cell.

[18] Brehme M., Voisine C., Rolland T. (2014). A chaperome subnetwork safeguards proteostasis in aging and neurodegenerative disease. Cell Rep.

[19] Shelton L.B., Koren J., Blair L.J. (2017). Imbalances in the Hsp90 chaperone machinery: implications for tauopathies. Front Neurosci.

[20] Kasza A., Hunya A., Frank Z. (2016). Dihydropyridine derivatives modulate heat shock responses and have a neuroprotective effect in a transgenic mouse model of Alzheimer's disease. J Alzheimers Dis.

[21] Jeanne X., Torok Z., Vigh L., Prodromou C. (2024). The role of the FKBP51-Hsp90 complex in Alzheimer's disease: an emerging new drug target. Cell Stress Chaperones.

[22] Oroz J., Chang B.J., Wysoczanski P. (2018). Structure and pro-toxic mechanism of the human Hsp90/PPIase/Tau complex. Nat Commun.

[23] Oberoi J., Guiu X.A., Outwin E.A. (2022). HSP90-CDC37-PP5 forms a structural platform for kinase dephosphorylation. Nat Commun.

[24] Gong C.X., Liu F., Wu G. (2004). Dephosphorylation of microtubule-associated protein tau by protein phosphatase 5. J Neurochem.

[25] Jinwal U.K., Koren J., Borysov S.I. (2010). The Hsp90 cochaperone, FKBP51, increases Tau stability and polymerizes microtubules. J Neurosci.

[26] Zhou X.Z., Kops O., Werner A. (2000). Pin1-dependent prolyl isomerization regulates dephosphorylation of Cdc25C and tau proteins. Mol Cell.

[27] Ma S.L., Pastorino L., Zhou X.Z., Lu K.P. (2012). Prolyl isomerase Pin1 promotes amyloid precursor protein (APP) turnover by inhibiting glycogen synthase kinase-3beta (GSK3beta) activity: novel mechanism for Pin1 to protect against Alzheimer disease. J Biol Chem.

[28] Liou Y.C., Sun A., Ryo A. (2003). Role of the prolyl isomerase Pin1 in protecting against age-dependent neurodegeneration. Nature.

[29] Patrick G.N., Zukerberg L., Nikolic M., de la Monte S., Dikkes P., Tsai L.H. (1999). Conversion of p35 to p25 deregulates Cdk5 activity and promotes neurodegeneration. Nature.

[30] Blair L.J., Nordhues B.A., Hill S.E. (2013). Accelerated neurodegeneration through chaperone-mediated oligomerization of tau. J Clin Invest.

[31] Sabbagh J.J., O'Leary J.C., Blair L.J. (2014). Age-associated epigenetic upregulation of the FKBP5 gene selectively impairs stress resiliency. PloS One.

[32] Sharma A., Srivastava S., Gupta P. (2025). Targeting protein misfolding in Alzheimer's disease: the emerging role of molecular chaperones. Biomed Pharmacother.

[33] Blair L.J., Baker J.D., Sabbagh J.J., Dickey C.A. (2015). The emerging role of peptidyl-prolyl isomerase chaperones in Tau oligomerization, amyloid processing, and Alzheimer's disease. J Neurochem.

[34] Lee K., Thwin A.C., Nadel C.M. (2021). The structure of an Hsp90-immunophilin complex reveals cochaperone recognition of the client maturation state. Mol Cell.

[35] Panaretou B., Prodromou C., Roe S.M. (1998). ATP binding and hydrolysis are essential to the function of the Hsp90 molecular chaperone in vivo. EMBO J.

[36] Panaretou B., Siligardi G., Meyer P. (2002). Activation of the ATPase activity of hsp90 by the stress-regulated cochaperone aha1. Mol Cell.

[37] Ali M.M., Roe S.M., Vaughan C.K. (2006). Crystal structure of an Hsp90-nucleotide-p23/Sba1 closed chaperone complex. Nature.

[38] Zhang M., Windheim M., Roe S.M. (2005). Chaperoned ubiquitylation--crystal structures of the CHIP U box E3 ubiquitin ligase and a CHIP-Ubc13-Uev1a complex. Mol Cell.

[39] Prodromou C., Siligardi G., O'Brien R. (1999). Regulation of Hsp90 ATPase activity by tetratricopeptide repeat (TPR)-domain co-chaperones. Embo J.

[40] Siligardi G., Panaretou B., Meyer P. (2002). Regulation of Hsp90 ATPase activity by the co-chaperone Cdc37p/p50^cdc37^. J Biol Chem.

[41] Oberoi J., Dunn D.M., Woodford M.R. (2016). Structural and functional basis of protein phosphatase 5 substrate specificity. Proc Natl Acad Sci U S A.

[42] Zhang M., Boter M., Li K. (2008). Structural and functional coupling of Hsp90- and Sgt1-centred multi-protein complexes. EMBO J.

[43] Eberhardt J., Santos-Martins D., Tillack A.F., Forli S. (2021). AutoDock Vina 1.2.0: new docking methods, expanded force field, and python bindings. J Chem Inf Model.

[44] Trott O., Olson A.J. (2010). AutoDock Vina: improving the speed and accuracy of docking with a new scoring function, efficient optimization, and multithreading. J Comput Chem.

[45] Yang J., Zhang Y. (2015). I-TASSER server: new development for protein structure and function predictions. Nucleic Acids Res.

[46] Zhang C., Mortuza S.M., He B., Wang Y., Zhang Y. (2018). Template-based and free modeling of I-TASSER and QUARK pipelines using predicted contact maps in CASP12. Proteins.

[47] Roe S.M., Ali M.M., Meyer P. (2004). The mechanism of Hsp90 regulation by the protein kinase-specific cochaperone p50(cdc37). Cell.

[48] Wang R.Y., Noddings C.M., Kirschke E., Myasnikov A.G., Johnson J.L., Agard D.A. (2022). Structure of Hsp90-Hsp70-Hop-GR reveals the Hsp90 client-loading mechanism. Nature.

[49] Zhang M., Kadota Y., Prodromou C., Shirasu K., Pearl L.H. (2010). Structural basis for assembly of Hsp90-Sgt1-CHORD protein complexes: implications for chaperoning of NLR innate immunity receptors. Mol Cell.

[50] Jaime-Garza M., Nowotny C.A., Coutandin D., Wang F., Tabios M., Agard D.A. (2023). Hsp90 provides a platform for kinase dephosphorylation by PP5. Nat Commun.

[51] Meduri G., Guillemeau K., Dounane O. (2016). Caspase-cleaved Tau-D(421) is colocalized with the immunophilin FKBP52 in the autophagy-endolysosomal system of Alzheimer's disease neurons. Neurobiol Aging.

[52] Liu F., Iqbal K., Grundke-Iqbal I., Rossie S., Gong C.X. (2005). Dephosphorylation of tau by protein phosphatase 5: impairment in Alzheimer's disease. J Biol Chem.

[53] Loerch P.M., Lu T., Dakin K.A. (2008). Evolution of the aging brain transcriptome and synaptic regulation. PloS one.

[54] Batko J., Antosz K., Miskow W., Pszczolowska M., Walczak K., Leszek J. (2024). Chaperones-a new class of potential therapeutic targets in Alzheimer's disease. Int J Mol Sci.

[55] Blair L.J., Sabbagh J.J., Dickey C.A. (2014). Targeting Hsp90 and its co-chaperones to treat Alzheimer's disease. Expert Opin Ther Targets.

[56] Shelton L.B., Baker J.D., Zheng D. (2017). Hsp90 activator Aha1 drives production of pathological tau aggregates. Proc Natl Acad Sci U S A.

[57] Criado-Marrero M., Gebru N.T., Blazier D.M. (2021). Hsp90 co-chaperones, FKBP52 and Aha1, promote tau pathogenesis in aged wild-type mice. Acta Neuropathol Commun.

