# Supplementary material for: The dihydropyridine LA1011 modulates multiple Hsp90—co-chaperone interactions relevant to Alzheimer’s disease
Source: Cell Stress Chaperones. 2025 Dec 3;31(1):100131. doi: 10.1016/j.cstres.2025.100131 (PMC12757481; doi:10.1016/j.cstres.2025.100131)
Supplement: Supplementary file 1 — Supplementary Material Figure 1. Paired sample T-test statistical analysis of ATPase assays. Statistical analysis for (a) Aha1; (b) FKBP51; (c) Sti1; (d) Sgt1 1–280; (e) CDC37; (f) p23; (g) CHIP; and (h) PP5. Confidence levels are indicated by one or more asterixis. (*) P value < 0.5, significant; (**) P value < 0.01, very significant and (***) P value < 0.001, highly significant [file mmc1.pptx]

## Slide 1
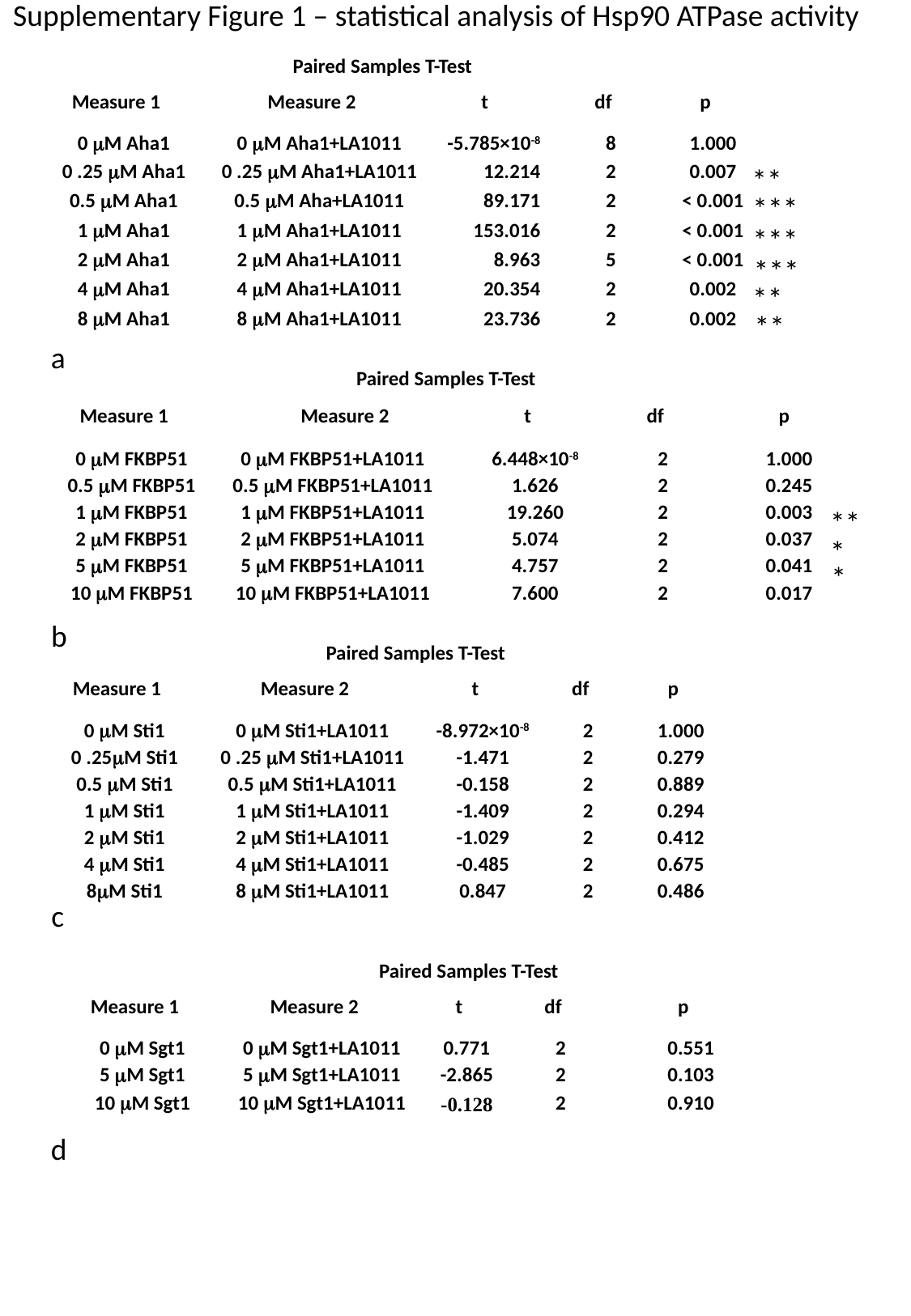

Supplementary Figure 1 – statistical analysis of Hsp90 ATPase activity
| Paired Samples T-Test | | | | |
| --- | --- | --- | --- | --- |
| Measure 1 | Measure 2 | t | df | p |
| 0 mM Aha1 | 0 mM Aha1+LA1011 | -5.785×10^-8^ | 8 | 1.000 |
| 0 .25 mM Aha1 | 0 .25 mM Aha1+LA1011 | 12.214 | 2 | 0.007 |
| 0.5 mM Aha1 | 0.5 mM Aha+LA1011 | 89.171 | 2 | < 0.001 |
| 1 mM Aha1 | 1 mM Aha1+LA1011 | 153.016 | 2 | < 0.001 |
| 2 mM Aha1 | 2 mM Aha1+LA1011 | 8.963 | 5 | < 0.001 |
| 4 mM Aha1 | 4 mM Aha1+LA1011 | 20.354 | 2 | 0.002 |
| 8 mM Aha1 | 8 mM Aha1+LA1011 | 23.736 | 2 | 0.002 |
**
***
***
***
**
**
a
| Paired Samples T-Test | | | | | | | | | | |
| --- | --- | --- | --- | --- | --- | --- | --- | --- | --- | --- |
| Measure 1 | Measure 2 | | t | df | | | | | p | |
| 0 mM FKBP51 | 0 mM FKBP51+LA1011 | 6.448×10^-8^ | | | | 2 | | 1.000 | | |
| 0.5 mM FKBP51 | 0.5 mM FKBP51+LA1011 | 1.626 | | | | 2 | | 0.245 | | |
| 1 mM FKBP51 | 1 mM FKBP51+LA1011 | 19.260 | | | | 2 | | 0.003 | | |
| 2 mM FKBP51 | 2 mM FKBP51+LA1011 | 5.074 | | | | 2 | | 0.037 | | |
| 5 mM FKBP51 | 5 mM FKBP51+LA1011 | 4.757 | | | | 2 | | 0.041 | | |
| 10 mM FKBP51 | 10 mM FKBP51+LA1011 | 7.600 | | | | 2 | | 0.017 | | |
| | | | | | | | | | | |
| | | | | | | | | | | |
**
*
*
b
| Paired Samples T-Test | | | | |
| --- | --- | --- | --- | --- |
| Measure 1 | Measure 2 | t | df | p |
| 0 mM Sti1 | 0 mM Sti1+LA1011 | -8.972×10^-8^ | 2 | 1.000 |
| 0 .25mM Sti1 | 0 .25 mM Sti1+LA1011 | -1.471 | 2 | 0.279 |
| 0.5 mM Sti1 | 0.5 mM Sti1+LA1011 | -0.158 | 2 | 0.889 |
| 1 mM Sti1 | 1 mM Sti1+LA1011 | -1.409 | 2 | 0.294 |
| 2 mM Sti1 | 2 mM Sti1+LA1011 | -1.029 | 2 | 0.412 |
| 4 mM Sti1 | 4 mM Sti1+LA1011 | -0.485 | 2 | 0.675 |
| 8mM Sti1 | 8 mM Sti1+LA1011 | 0.847 | 2 | 0.486 |
| | | | | |
| | | | | |
c
| Paired Samples T-Test | | | | |
| --- | --- | --- | --- | --- |
| Measure 1 | Measure 2 | t | df | p |
| 0 mM Sgt1 | 0 mM Sgt1+LA1011 | 0.771 | 2 | 0.551 |
| 5 mM Sgt1 | 5 mM Sgt1+LA1011 | -2.865 | 2 | 0.103 |
| 10 mM Sgt1 | 10 mM Sgt1+LA1011 | -0.128 | 2 | 0.910 |
| | | | | |
| | | | | |
d

## Slide 2
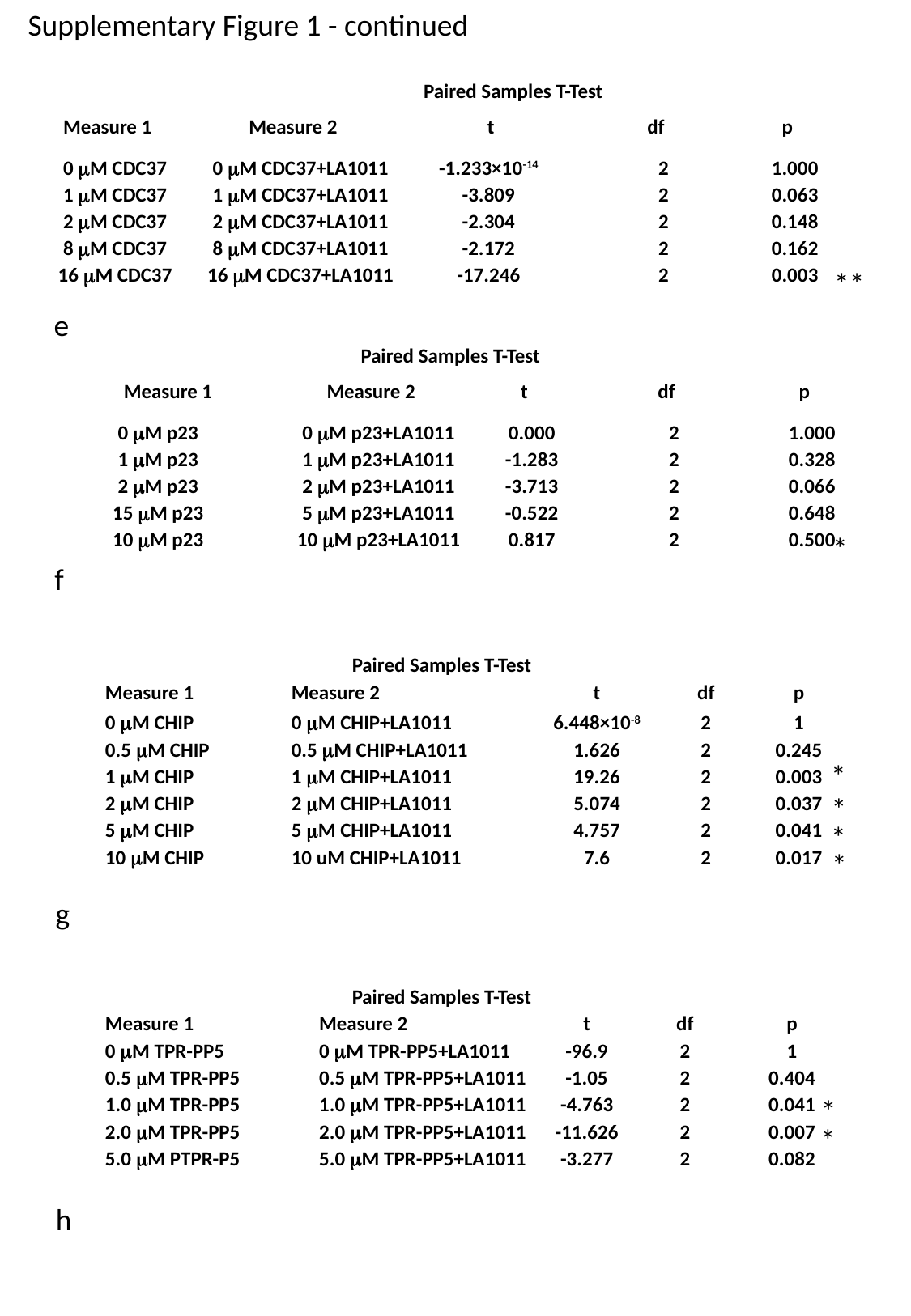

Supplementary Figure 1 - continued
| Paired Samples T-Test | | | | | |
| --- | --- | --- | --- | --- | --- |
| Measure 1 | Measure 2 | t | | df | p |
| 0 mM CDC37 | 0 mM CDC37+LA1011 | -1.233×10^-14^ | | 2 | 1.000 |
| 1 mM CDC37 | 1 mM CDC37+LA1011 | -3.809 | | 2 | 0.063 |
| 2 mM CDC37 | 2 mM CDC37+LA1011 | -2.304 | | 2 | 0.148 |
| 8 mM CDC37 | 8 mM CDC37+LA1011 | -2.172 | | 2 | 0.162 |
| 16 mM CDC37 | 16 mM CDC37+LA1011 | -17.246 | | 2 | 0.003 |
| | | | | | |
| | | | | | |
**
e
| Paired Samples T-Test | | | | | |
| --- | --- | --- | --- | --- | --- |
| Measure 1 | | Measure 2 | t | df | p |
| 0 mM p23 | | 0 mM p23+LA1011 | 0.000 | 2 | 1.000 |
| 1 mM p23 | | 1 mM p23+LA1011 | -1.283 | 2 | 0.328 |
| 2 mM p23 | | 2 mM p23+LA1011 | -3.713 | 2 | 0.066 |
| 15 mM p23 | | 5 mM p23+LA1011 | -0.522 | 2 | 0.648 |
| 10 mM p23 | | 10 mM p23+LA1011 | 0.817 | 2 | 0.500 |
| | | | | | |
| | | | | | |
*
f
| Paired Samples T-Test | | | | | |
| --- | --- | --- | --- | --- | --- |
| Measure 1 | | Measure 2 | t | df | p |
| 0 mM CHIP | | 0 mM CHIP+LA1011 | 6.448×10^-8^ | 2 | 1 |
| 0.5 mM CHIP | | 0.5 mM CHIP+LA1011 | 1.626 | 2 | 0.245 |
| 1 mM CHIP | | 1 mM CHIP+LA1011 | 19.26 | 2 | 0.003 |
| 2 mM CHIP | | 2 mM CHIP+LA1011 | 5.074 | 2 | 0.037 |
| 5 mM CHIP | | 5 mM CHIP+LA1011 | 4.757 | 2 | 0.041 |
| 10 mM CHIP | | 10 uM CHIP+LA1011 | 7.6 | 2 | 0.017 |
| | | | | | |
| | | | | | |
*
*
*
*
g
| Paired Samples T-Test | | | | | | |
| --- | --- | --- | --- | --- | --- | --- |
| Measure 1 | | | Measure 2 | t | df | p |
| 0 mM TPR-PP5 | | | 0 mM TPR-PP5+LA1011 | -96.9 | 2 | 1 |
| 0.5 mM TPR-PP5 | | | 0.5 mM TPR-PP5+LA1011 | -1.05 | 2 | 0.404 |
| 1.0 mM TPR-PP5 | | | 1.0 mM TPR-PP5+LA1011 | -4.763 | 2 | 0.041 |
| 2.0 mM TPR-PP5 | | | 2.0 mM TPR-PP5+LA1011 | -11.626 | 2 | 0.007 |
| 5.0 mM PTPR-P5 | | | 5.0 mM TPR-PP5+LA1011 | -3.277 | 2 | 0.082 |
| | | | | | | |
| | | | | | | |
*
*
h
